# 1-(1-Nonyl-2-oxoindolin-3-yl­idene)thio­semicarbazide

**DOI:** 10.1107/S160053681001651X

**Published:** 2010-05-15

**Authors:** Khalil Mamari, Abdelfettah Zerzouf, Hafid Zouihri, El Mokhtar Essassi, Seik Weng Ng

**Affiliations:** a Laboratoire de Chimie Organique Hétérocyclique, Pôle de Compétences Pharmacochimie, Université Mohammed V-Agdal, BP 1014 Avenue Ibn Batout, Rabat, Morocco; b Laboratoire de Chimie Organique et Etudes Physicochimiques, ENS Rabat, Morocco; c CNRST Division UATRS, Angle Allal Fassi/FAR, BP 8027 Hay Riad, 0000 Rabat, Morocco; d Department of Chemistry, University of Malaya, 50603 Kuala Lumpur, Malaysia

## Abstract

In the title compound, C_18_H_26_N_4_OS, the imine C=N bond has a *Z* configuration, whereas the N—N—C=S unit has an *E* conformation. In the crystal, mol­ecules are connected through N—H⋯O hydrogen bonds, forming zigzag chains running along the *b* axis. The nonyl chain adopts an extended zigzag conformation.

## Related literature

For background to *N*-substituted isatins and their derivatives, see: Bouhfid *et al.* (2008 ▶).
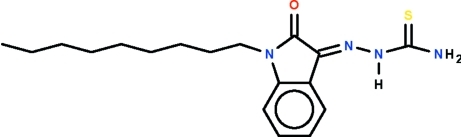


## Experimental

### 

#### Crystal data

C_18_H_26_N_4_OS
*M* *_r_* = 346.49
Monoclinic, 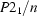

*a* = 11.5343 (2) Å
*b* = 10.5921 (2) Å
*c* = 15.6262 (3) Å
β = 95.922 (1)°
*V* = 1898.90 (6) Å^3^
*Z* = 4
Mo *K*α radiation
μ = 0.18 mm^−1^
*T* = 180 K
0.17 × 0.15 × 0.09 mm

#### Data collection

Bruker X8 APEXII diffractometer
Absorption correction: multi-scan (*SADABS*; Sheldrick, 1996 ▶) *T*_min_ = 0.970, *T*_max_ = 0.984
26603 measured reflections
5510 independent reflections
4006 reflections with *I* > 2σ(*I*)
*R*_int_ = 0.041

#### Refinement

*R*[*F* ^2^ > 2σ(*F* ^2^)] = 0.041
*wR*(*F* ^2^) = 0.134
*S* = 1.09
5510 reflections
229 parameters
3 restraints
H atoms treated by a mixture of independent and constrained refinement
Δρ_max_ = 0.48 e Å^−3^
Δρ_min_ = −0.51 e Å^−3^

### 

Data collection: *APEX2* (Bruker, 2008 ▶); cell refinement: *SAINT* (Bruker, 2008 ▶); data reduction: *SAINT*; program(s) used to solve structure: *SHELXS97* (Sheldrick, 2008 ▶); program(s) used to refine structure: *SHELXL97* (Sheldrick, 2008 ▶); molecular graphics: *X-SEED* (Barbour, 2001 ▶); software used to prepare material for publication: *publCIF* (Westrip, 2010 ▶).

## Supplementary Material

Crystal structure: contains datablocks global, I. DOI: 10.1107/S160053681001651X/bt5264sup1.cif

Structure factors: contains datablocks I. DOI: 10.1107/S160053681001651X/bt5264Isup2.hkl

Additional supplementary materials:  crystallographic information; 3D view; checkCIF report

## Figures and Tables

**Table 1:** Hydrogen-bond geometry (Å, °)

*D*—H⋯*A*	*D*—H	H⋯*A*	*D*⋯*A*	*D*—H⋯*A*
N1—H12⋯O1^i^	0.86 (1)	2.22 (1)	3.002 (2)	152 (2)

Symmetry code: (i) 
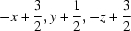
.

**Fig. 1.**
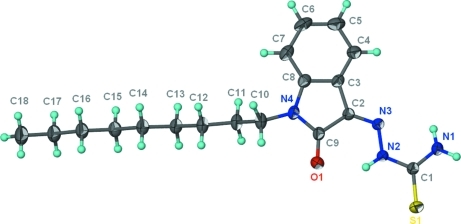
Thermal ellipsoid plot (Barbour, 2001) of the molecule of C18H26N4OS at the 70% probability level; hydrogen atoms are drawn as spheres of arbitrary radius.

**Fig. 2.**
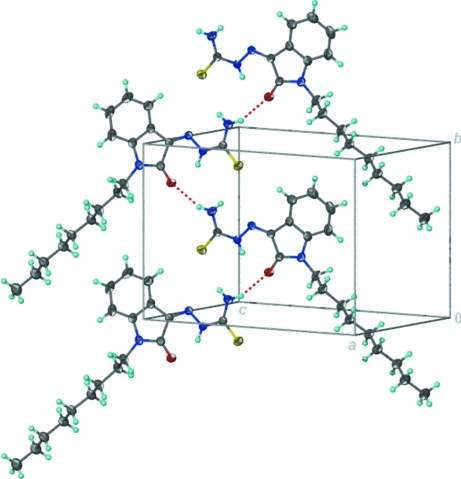
Hydrogen-bonded helical chain motif.

## supplementary crystallographic information

### Comment

*N*-Substituted isatins (Bouhfid *et al.*, 2008) represent a large family of heterocyclic compounds reported to show a wide range of useful medicinal properties. These condense readily with thiosemicarbazides to form crystalline thiosemicarbazones. The title 1-nonylisatin derivative has a *Z* configuration around the imine C=N bond and *E* conformation about the C(=S)–NH_2_ bond (Scheme I, Fig. 1). The molecules are connected to zigzag chains though N–H···O hydrogen bonds along the *b*-axis of the monoclinic unit cell (Fig. 2). The nonyl chain adopts a nearly regular zigzag conformation; however, the rigid nature gives rises to voids in the crystal.

### Experimental

1-Nonyl-isatin (1 g ,3 mmol) and thiosemicarbazide (0.27 g ,3 mmol) were dissolved in aqueous ethanol (50 ml); a few drops of glacial acetic acid were added. The mixture was heated for 4 hours. Yellow crystals separated from the cool solution in 80% yield.

### Refinement

Carbon-bound H-atoms were placed in calculated positions (C—H 0.95–0.99 Å) and were included in the refinement in the riding model approximation, with *U*(H) set to 1.2–1.5*U*_eq_(C). The amino-H atoms were located in a difference Fourier map, and they were refined isotropically with a distance restraint of N–H 0.86±0.01 Å.

### Crystal data

**Table:**

C_18_H_26_N_4_OS	*F*(000) = 744
*M**_r_* = 346.49	*D*_x_ = 1.212 Mg m^−^^3^
Monoclinic, *P*2_1_/*n*	Mo *K*α radiation, λ = 0.71073 Å
Hall symbol: -P 2yn	Cell parameters from 6721 reflections
*a* = 11.5343 (2) Å	θ = 2.6–29.8°
*b* = 10.5921 (2) Å	µ = 0.18 mm^−^^1^
*c* = 15.6262 (3) Å	*T* = 180 K
β = 95.922 (1)°	Prism, yellow
*V* = 1898.90 (6) Å^3^	0.17 × 0.15 × 0.09 mm
*Z* = 4	

### Data collection

**Table:**

Bruker X8 APEXII diffractometer	5510 independent reflections
Radiation source: fine-focus sealed tube	4006 reflections with *I* > 2σ(*I*)
graphite	*R*_int_ = 0.041
φ and ω scans	θ_max_ = 30.0°, θ_min_ = 2.1°
Absorption correction: multi-scan (*SADABS*; Sheldrick, 1996)	*h* = −16→16
*T*_min_ = 0.970, *T*_max_ = 0.984	*k* = −14→14
26603 measured reflections	*l* = −21→21

### Refinement

**Table:**

Refinement on *F*^2^	Primary atom site location: structure-invariant direct methods
Least-squares matrix: full	Secondary atom site location: difference Fourier map
*R*[*F*^2^ > 2σ(*F*^2^)] = 0.041	Hydrogen site location: inferred from neighbouring sites
*wR*(*F*^2^) = 0.134	H atoms treated by a mixture of independent and constrained refinement
*S* = 1.09	*w* = 1/[σ^2^(*F*_o_^2^) + (0.0722*P*)^2^ + 0.1904*P*] where *P* = (*F*_o_^2^ + 2*F*_c_^2^)/3
5510 reflections	(Δ/σ)_max_ = 0.001
229 parameters	Δρ_max_ = 0.48 e Å^−^^3^
3 restraints	Δρ_min_ = −0.50 e Å^−^^3^

### Special details

**Table:**

Geometry. All esds (except the esd in the dihedral angle between two l.s. planes) are estimated using the full covariance matrix. The cell esds are taken into account individually in the estimation of esds in distances, angles and torsion angles; correlations between esds in cell parameters are only used when they are defined by crystal symmetry. An approximate (isotropic) treatment of cell esds is used for estimating esds involving l.s. planes.

### Fractional atomic coordinates and isotropic or equivalent isotropic displacement parameters (Å^2^)

**Table:**

	*x*	*y*	*z*	*U*_iso_*/*U*_eq_	
S1	0.76947 (4)	0.88269 (3)	0.65446 (2)	0.02943 (12)	
O1	0.86020 (10)	0.77068 (9)	0.93146 (7)	0.0283 (2)	
N1	0.73497 (14)	1.11116 (12)	0.71834 (9)	0.0314 (3)	
N2	0.78603 (11)	0.95357 (11)	0.81532 (8)	0.0230 (3)	
N3	0.79185 (10)	1.03853 (11)	0.88032 (7)	0.0213 (2)	
N4	0.90309 (11)	0.86435 (11)	1.06470 (8)	0.0240 (3)	
C1	0.76202 (13)	0.99075 (13)	0.73160 (9)	0.0221 (3)	
C2	0.82742 (12)	0.99591 (12)	0.95604 (9)	0.0205 (3)	
C3	0.84765 (12)	1.07018 (14)	1.03435 (9)	0.0224 (3)	
C4	0.83255 (13)	1.19707 (14)	1.05261 (10)	0.0276 (3)	
H4	0.8005	1.2539	1.0095	0.033*	
C5	0.86590 (15)	1.23851 (16)	1.13619 (11)	0.0350 (4)	
H5	0.8562	1.3248	1.1503	0.042*	
C6	0.91290 (15)	1.15564 (17)	1.19886 (11)	0.0360 (4)	
H6	0.9352	1.1865	1.2553	0.043*	
C7	0.92842 (14)	1.02816 (16)	1.18136 (10)	0.0304 (3)	
H7	0.9605	0.9716	1.2247	0.037*	
C8	0.89532 (12)	0.98741 (14)	1.09875 (9)	0.0237 (3)	
C9	0.86411 (12)	0.86286 (13)	0.97964 (9)	0.0221 (3)	
C10	0.95911 (14)	0.75743 (14)	1.11096 (10)	0.0283 (3)	
H10A	1.0326	0.7868	1.1432	0.034*	
H10B	0.9797	0.6939	1.0686	0.034*	
C11	0.88473 (13)	0.69389 (14)	1.17376 (10)	0.0275 (3)	
H11A	0.8186	0.6493	1.1415	0.033*	
H11B	0.8527	0.7585	1.2106	0.033*	
C12	0.95880 (13)	0.60010 (14)	1.22971 (10)	0.0281 (3)	
H12A	0.9906	0.5368	1.1918	0.034*	
H12B	1.0256	0.6459	1.2601	0.034*	
C13	0.89450 (13)	0.53100 (14)	1.29607 (9)	0.0260 (3)	
H13A	0.8586	0.5936	1.3323	0.031*	
H13B	0.8312	0.4795	1.2661	0.031*	
C14	0.97559 (13)	0.44591 (14)	1.35311 (10)	0.0265 (3)	
H14A	1.0372	0.4986	1.3841	0.032*	
H14B	1.0140	0.3865	1.3161	0.032*	
C15	0.91596 (13)	0.36994 (14)	1.41887 (10)	0.0273 (3)	
H15A	0.8789	0.4290	1.4570	0.033*	
H15B	0.8536	0.3179	1.3883	0.033*	
C16	0.99913 (14)	0.28424 (14)	1.47370 (10)	0.0289 (3)	
H16A	1.0606	0.3368	1.5050	0.035*	
H16B	1.0374	0.2268	1.4353	0.035*	
C17	0.94113 (16)	0.20532 (16)	1.53871 (11)	0.0360 (4)	
H17A	0.8791	0.1531	1.5078	0.043*	
H17B	0.9040	0.2624	1.5780	0.043*	
C18	1.0270 (2)	0.11952 (18)	1.59148 (13)	0.0487 (5)	
H18A	0.9857	0.0709	1.6322	0.073*	
H18B	1.0877	0.1709	1.6232	0.073*	
H18C	1.0628	0.0616	1.5530	0.073*	
H11	0.7307 (17)	1.1607 (16)	0.7620 (9)	0.043 (5)*	
H12	0.7173 (18)	1.1345 (19)	0.6657 (7)	0.050 (6)*	
H2	0.8141 (15)	0.8786 (11)	0.8267 (12)	0.036 (5)*	

### Atomic displacement parameters (Å^2^)

**Table:**

	*U*^11^	*U*^22^	*U*^33^	*U*^12^	*U*^13^	*U*^23^
S1	0.0426 (2)	0.02504 (19)	0.02068 (19)	−0.00110 (15)	0.00317 (16)	−0.00439 (13)
O1	0.0369 (6)	0.0213 (5)	0.0260 (6)	−0.0001 (4)	0.0002 (5)	−0.0006 (4)
N1	0.0521 (9)	0.0226 (6)	0.0189 (6)	0.0049 (6)	0.0001 (6)	0.0012 (5)
N2	0.0315 (7)	0.0189 (6)	0.0177 (6)	0.0004 (5)	−0.0013 (5)	−0.0006 (4)
N3	0.0246 (6)	0.0207 (5)	0.0183 (6)	−0.0014 (4)	0.0014 (5)	−0.0005 (4)
N4	0.0269 (6)	0.0258 (6)	0.0189 (6)	0.0009 (5)	0.0005 (5)	0.0040 (4)
C1	0.0254 (7)	0.0213 (6)	0.0192 (7)	−0.0022 (5)	0.0011 (5)	0.0009 (5)
C2	0.0221 (7)	0.0205 (6)	0.0190 (7)	−0.0012 (5)	0.0023 (5)	0.0006 (5)
C3	0.0232 (7)	0.0263 (7)	0.0177 (6)	−0.0021 (5)	0.0022 (5)	−0.0004 (5)
C4	0.0314 (8)	0.0264 (7)	0.0252 (7)	−0.0015 (6)	0.0031 (6)	−0.0029 (6)
C5	0.0416 (9)	0.0334 (8)	0.0304 (9)	−0.0051 (7)	0.0060 (7)	−0.0108 (6)
C6	0.0385 (9)	0.0475 (10)	0.0217 (8)	−0.0080 (7)	0.0020 (7)	−0.0104 (7)
C7	0.0301 (8)	0.0400 (9)	0.0207 (7)	−0.0038 (6)	0.0001 (6)	0.0011 (6)
C8	0.0224 (7)	0.0283 (7)	0.0205 (7)	−0.0033 (5)	0.0026 (5)	0.0005 (5)
C9	0.0228 (7)	0.0232 (6)	0.0202 (7)	−0.0022 (5)	0.0013 (5)	0.0033 (5)
C10	0.0287 (8)	0.0303 (7)	0.0256 (8)	0.0039 (6)	0.0019 (6)	0.0090 (6)
C11	0.0277 (8)	0.0293 (7)	0.0253 (7)	0.0007 (6)	0.0016 (6)	0.0084 (6)
C12	0.0280 (8)	0.0306 (7)	0.0255 (8)	0.0026 (6)	0.0025 (6)	0.0086 (6)
C13	0.0273 (7)	0.0275 (7)	0.0234 (7)	0.0012 (6)	0.0030 (6)	0.0037 (5)
C14	0.0273 (7)	0.0276 (7)	0.0248 (7)	0.0014 (6)	0.0040 (6)	0.0055 (6)
C15	0.0288 (8)	0.0276 (7)	0.0261 (7)	−0.0001 (6)	0.0061 (6)	0.0051 (6)
C16	0.0330 (8)	0.0279 (7)	0.0263 (8)	0.0014 (6)	0.0057 (6)	0.0061 (6)
C17	0.0444 (10)	0.0335 (8)	0.0298 (8)	−0.0051 (7)	0.0029 (7)	0.0092 (7)
C18	0.0659 (13)	0.0398 (10)	0.0372 (10)	−0.0097 (9)	−0.0100 (9)	0.0145 (8)

### Geometric parameters (Å, °)

**Table:**

S1—C1	1.6710 (14)	C10—H10B	0.9900
O1—C9	1.2309 (17)	C11—C12	1.525 (2)
N1—C1	1.3242 (19)	C11—H11A	0.9900
N1—H11	0.866 (9)	C11—H11B	0.9900
N1—H12	0.862 (9)	C12—C13	1.523 (2)
N2—N3	1.3534 (16)	C12—H12A	0.9900
N2—C1	1.3669 (18)	C12—H12B	0.9900
N2—H2	0.869 (9)	C13—C14	1.520 (2)
N3—C2	1.2931 (17)	C13—H13A	0.9900
N4—C9	1.3585 (18)	C13—H13B	0.9900
N4—C8	1.4142 (18)	C14—C15	1.524 (2)
N4—C10	1.4582 (18)	C14—H14A	0.9900
C2—C3	1.4532 (19)	C14—H14B	0.9900
C2—C9	1.5063 (19)	C15—C16	1.519 (2)
C3—C4	1.389 (2)	C15—H15A	0.9900
C3—C8	1.403 (2)	C15—H15B	0.9900
C4—C5	1.394 (2)	C16—C17	1.523 (2)
C4—H4	0.9500	C16—H16A	0.9900
C5—C6	1.384 (3)	C16—H16B	0.9900
C5—H5	0.9500	C17—C18	1.522 (3)
C6—C7	1.393 (2)	C17—H17A	0.9900
C6—H6	0.9500	C17—H17B	0.9900
C7—C8	1.377 (2)	C18—H18A	0.9800
C7—H7	0.9500	C18—H18B	0.9800
C10—C11	1.525 (2)	C18—H18C	0.9800
C10—H10A	0.9900		
			
C1—N1—H11	119.5 (14)	C10—C11—H11B	109.7
C1—N1—H12	117.0 (14)	C12—C11—H11B	109.7
H11—N1—H12	123.4 (19)	H11A—C11—H11B	108.2
N3—N2—C1	121.07 (11)	C13—C12—C11	114.83 (13)
N3—N2—H2	117.7 (13)	C13—C12—H12A	108.6
C1—N2—H2	119.6 (13)	C11—C12—H12A	108.6
C2—N3—N2	116.17 (11)	C13—C12—H12B	108.6
C9—N4—C8	110.57 (11)	C11—C12—H12B	108.6
C9—N4—C10	124.13 (12)	H12A—C12—H12B	107.5
C8—N4—C10	124.85 (12)	C14—C13—C12	111.77 (13)
N1—C1—N2	116.64 (13)	C14—C13—H13A	109.3
N1—C1—S1	125.15 (11)	C12—C13—H13A	109.3
N2—C1—S1	118.21 (10)	C14—C13—H13B	109.3
N3—C2—C3	126.14 (12)	C12—C13—H13B	109.3
N3—C2—C9	127.25 (12)	H13A—C13—H13B	107.9
C3—C2—C9	106.48 (12)	C13—C14—C15	114.55 (13)
C4—C3—C8	120.37 (13)	C13—C14—H14A	108.6
C4—C3—C2	133.18 (13)	C15—C14—H14A	108.6
C8—C3—C2	106.41 (12)	C13—C14—H14B	108.6
C3—C4—C5	117.97 (15)	C15—C14—H14B	108.6
C3—C4—H4	121.0	H14A—C14—H14B	107.6
C5—C4—H4	121.0	C16—C15—C14	113.15 (13)
C6—C5—C4	120.90 (15)	C16—C15—H15A	108.9
C6—C5—H5	119.6	C14—C15—H15A	108.9
C4—C5—H5	119.5	C16—C15—H15B	108.9
C5—C6—C7	121.67 (15)	C14—C15—H15B	108.9
C5—C6—H6	119.2	H15A—C15—H15B	107.8
C7—C6—H6	119.2	C15—C16—C17	114.13 (13)
C8—C7—C6	117.33 (15)	C15—C16—H16A	108.7
C8—C7—H7	121.3	C17—C16—H16A	108.7
C6—C7—H7	121.3	C15—C16—H16B	108.7
C7—C8—C3	121.77 (14)	C17—C16—H16B	108.7
C7—C8—N4	128.26 (14)	H16A—C16—H16B	107.6
C3—C8—N4	109.97 (12)	C16—C17—C18	112.53 (15)
O1—C9—N4	126.53 (13)	C16—C17—H17A	109.1
O1—C9—C2	126.92 (13)	C18—C17—H17A	109.1
N4—C9—C2	106.55 (12)	C16—C17—H17B	109.1
N4—C10—C11	114.32 (13)	C18—C17—H17B	109.1
N4—C10—H10A	108.7	H17A—C17—H17B	107.8
C11—C10—H10A	108.7	C17—C18—H18A	109.5
N4—C10—H10B	108.7	C17—C18—H18B	109.5
C11—C10—H10B	108.7	H18A—C18—H18B	109.5
H10A—C10—H10B	107.6	C17—C18—H18C	109.5
C10—C11—C12	109.71 (13)	H18A—C18—H18C	109.5
C10—C11—H11A	109.7	H18B—C18—H18C	109.5
C12—C11—H11A	109.7		
			
C1—N2—N3—C2	−172.75 (13)	C10—N4—C8—C7	−5.9 (2)
N3—N2—C1—N1	−5.7 (2)	C9—N4—C8—C3	1.03 (17)
N3—N2—C1—S1	173.97 (10)	C10—N4—C8—C3	173.50 (13)
N2—N3—C2—C3	175.91 (13)	C8—N4—C9—O1	179.53 (14)
N2—N3—C2—C9	0.7 (2)	C10—N4—C9—O1	7.0 (2)
N3—C2—C3—C4	2.9 (3)	C8—N4—C9—C2	−0.18 (16)
C9—C2—C3—C4	178.91 (16)	C10—N4—C9—C2	−172.71 (13)
N3—C2—C3—C8	−174.78 (14)	N3—C2—C9—O1	−4.4 (2)
C9—C2—C3—C8	1.27 (15)	C3—C2—C9—O1	179.61 (14)
C8—C3—C4—C5	−0.1 (2)	N3—C2—C9—N4	175.31 (14)
C2—C3—C4—C5	−177.47 (15)	C3—C2—C9—N4	−0.69 (15)
C3—C4—C5—C6	0.2 (2)	C9—N4—C10—C11	−108.71 (16)
C4—C5—C6—C7	−0.2 (3)	C8—N4—C10—C11	79.81 (18)
C5—C6—C7—C8	0.2 (2)	N4—C10—C11—C12	−169.81 (13)
C6—C7—C8—C3	−0.1 (2)	C10—C11—C12—C13	179.42 (13)
C6—C7—C8—N4	179.29 (15)	C11—C12—C13—C14	−176.25 (13)
C4—C3—C8—C7	0.1 (2)	C12—C13—C14—C15	−177.78 (13)
C2—C3—C8—C7	178.06 (14)	C13—C14—C15—C16	178.98 (13)
C4—C3—C8—N4	−179.43 (13)	C14—C15—C16—C17	−178.83 (13)
C2—C3—C8—N4	−1.42 (16)	C15—C16—C17—C18	179.22 (15)
C9—N4—C8—C7	−178.41 (15)		

### Hydrogen-bond geometry (Å, °)

**Table:**

*D*—H···*A*	*D*—H	H···*A*	*D*···*A*	*D*—H···*A*
N1—H11···S1^i^	0.87 (1)	2.69 (1)	3.499 (1)	156 (2)
N1—H12···O1^i^	0.86 (1)	2.22 (1)	3.002 (2)	152 (2)

Symmetry codes: (i) −*x*+3/2, *y*+1/2, −*z*+3/2.
